# Cultural adaptation experiences of long-term older Turkish immigrants: a qualitative study

**DOI:** 10.1186/s12912-025-03848-6

**Published:** 2025-09-26

**Authors:** Ayşegül Ilgaz, Büşra Nur Temür, Süleyman Şahin, Sebahat Gözüm, Anne-Sofie Helvik

**Affiliations:** 1 Faculty of Nursing, Department of Public Health Nursing, Akdeniz University, Antalya, Türkiye; 2 Faculty of Nursing, Department of Surgical Nursing, Akdeniz University, Antalya, Türkiye; 3 Faculty of Medicine and Health Sciences, Department of Public Health and Nursing, Norwegian University of Science and Technology (NTNU), Trondheim, Norway; 4 Faculty of Health Sciences, Department of Public Health Nursing, Kafkas University, Kars, Türkiye; 5 Norwegian National Centre for Ageing and Health, Vestfold Hospital Trust, Tønsberg, Norway

**Keywords:** Cultural adaptation, Facilitators, Barriers, Immigrant, Older people, Nursing, Public health nursing

## Abstract

**Background:**

It is of interest to local authorities to better understand cultural adaptation among immigrant groups who came to a new culture and country at a young age. There is a gap in the literature on the cultural adaptation of Turkish immigrants from arrival until and during old age. This study aimed to explore the experiences of cultural adaptation among long-term older Turkish immigrants in Norway.

**Methods:**

This study had a qualitative, explorative design and employed reflexive thematic analysis and face-to-face semi-structured individual interviews to improve the understanding of cultural adaptation. A total of 15 Turkish immigrants aged 60 years and over were interviewed once between March and June 2023.

**Results:**

The following two main themes and five subthemes about cultural adaptation were generated: (a) encouraging cultural adaptation (experiencing social equality, tolerance, and economic prosperity; meeting supportive people and functioning welfare systems; learning and liking the culture and people); (b) restricting cultural adaptation (a continuing language barrier: struggling to communicate; preserving one’s own culture and heritage).

**Conclusion:**

To enhance cultural adaptation, researchers should conduct interventional studies that consider encouragement and restriction. For long-term older migrants, nursing is an essential tool for enhancing cultural adaption and promoting healthy aging in the host country. This study recommends further education and research for nurses to be more effective in overcoming cultural barriers and supporting older immigrants.

**Clinical trial number:**

Not applicable.

**Supplementary Information:**

The online version contains supplementary material available at 10.1186/s12912-025-03848-6.

## Introduction

With the effect of globalization, there has been an increase in working areas and transportation opportunities, as well as technological developments. In addition, there has been geographical change due to situations such as war, natural disasters, better education, and a more comfortable life [1]. The mobility that occurred in this process revealed the concept of multiculturalism. Almost all societies have experienced changes in their cultural structures, which were previously based on a single culture, language, or identity [2].

There are several reasons why people leave their homelands and migrate. Individuals migrate when they move for reasons such as better living conditions, improved access to education, job opportunities, or safety [3]. Through this movement, they bring their cultural, social, and economic values into a new environment. Migration leads to changes in the demographic composition of society, an increase in cultural diversity, and the development of social adaptations. However, it can also lead to the redistribution of resources, social conflicts, and difficulties with integration [4]. People may face challenges related to identity, issues with social cohesion, and changes in their lifestyles [5]. While immigrating individuals experience a process of cultural adaptation, countries receiving immigration are expected to develop implementation methods that will facilitate the coexistence and adaptation of individuals from different cultures [6].

Cultural adaptation is a dynamic process that involves members of cultural minorities adopting certain characteristics of the culture of the host country and maintaining their lives and involves both external and internal adjustments [7]. The process of cultural adaptation requires individuals to make changes in basic things, such as customs and traditions, celebrations, behavior, and eating habits [8]. Cultural adaptation is important because it encourages better participation in new environments. In addition, this adaptation helps individuals feel control over or ownership of the new domains. A balance between assimilation and cultural preservation is necessary to achieve well-being [2, 9, 10]. Cultural adaptation includes acculturation, which is embracing new cultural norms, values, and behaviors by interaction with the host society, and enculturation, which is the internalization of values, beliefs, and behaviors from one’s culture of origin since childhood [11, 12]. People can successfully integrate into the new culture while maintaining their identity thanks to this dual process. Increased social integration, a feeling of belonging, and personal development are possible outcomes for the individual; nevertheless, juggling two cultural frameworks may also cause stress, identity conflict, and emotional difficulties [13]. The literature demonstrates that immigrants’ psychological health, identity development, and social functioning are greatly impacted by cultural adaptation, which encompasses both enculturation and acculturation. Decreased acculturative stress and increased life satisfaction are linked to successfully integrating into the host culture while maintaining one’s original cultural identity [14–16]. One of the most widely used models, Berry’s acculturation framework, distinguishes four adjustment techniques based on how much a person adopts the host culture and preserves their heritage culture: Marginalization, integration, separation, and assimilation [17]. The majority of other theories, such The Melting Pot One-Dimensional Model and Kim’s integrative theory of cross-cultural adaptation, concentrate on ideas like a person’s cultural identity, psychological health, degree of social contact, social integration, and cultural conflicts [18, 19]. There are restrictions and encouragement in the cultural adaptation process of immigrants. Understanding these restrictions is important for ensuring equitable conditions among individuals in society [20]. In a study of immigrant individuals in Brazil, sociocultural adaptation was the only positive predictor of general well-being [21]. A recent study evaluating the adaptation, assimilation, and integration of immigrants reported that Romanian immigrants’ material and financial situations improved after migration. In addition, their relationships with their families and communities were strengthened, they participated in new social areas, and their professional and entrepreneurial skills improved [8]. The cultural adaptation process of immigrant individuals includes not only their adaptation to health services but also to social and community services and society.

Migration is a social movement in which causes and consequences are important [22, 23]. Migration not only changes the place where people live but also carries their material and spiritual values, lifestyle, beliefs, eating and drinking habits, traditions, and cultural past to the place where they migrate. Therefore, social, economic, and cultural effects occur as a result of migration [2, 24–26]. Türkiye citizens started to come to Norway for work from 1960 to 1975, along with citizens mostly from Yugoslavia and Pakistan. Later, citizens were coming from Türkiye, either as refugees asking for protection or as family members of immigrants or refugees. The number of first-generation immigrants in Norway is high, and there are 800,000 citizens but we do not divide them by age or nation [27].

The cultural adaptation process of immigrants is important in terms of continuing their lives healthily and happily in the host country [21, 28]. Nursing plays a vital role in developing culturally sensitive care strategies, making it important to understand the cultural adaptation processes of older migrants. According to Afaf Meleis’ Transition Theory, an esteemed nurse scientist, migration represents a significant situational transition that emphasizes cultural adaptation, impacting an individual’s environment, roles, relationships, and sense of identity. This transition entails a complex experience that requires individuals to maintain their cultural identity while also adjusting to a new society, which is particularly relevant for older migrants [29, 30]. The processes of enculturation and acculturation play critical roles in influencing the health, sense of belonging, and social roles of these individuals, and nursing care serves as a vital support system in navigating this transition. In this regard, culturally sensitive nursing practices are crucial in facilitating healthy aging and the integration of older immigrants into their new communities [31, 32].

Long-term immigrants may have a harder time adjusting to a new culture as they get older, particularly if they have a stronger yearning for their own country. This should be taken into consideration when evaluating their emotional and psychological requirements. To promote the well-being of older Turkish migrants and foster healthier aging, nurses need to possess a deep understanding of their specific health and cultural requirements. Nursing plays a fundamental role in supporting the cultural adaptation and healthy ageing of older migrants, and nurses are a key resource in ensuring that migrants feel valued and have access to health services [33, 34].

Throughout old age, they may have difficulty adapting to the lifestyle of the country they migrated to at a young age [35, 36]. This study concentrates on older Turkish immigrants in Norway because, despite having lived as immigrants for many years, they encounter fresh obstacles in the cultural adaptation process as they age. Younger immigrants experience more severe problems with belonging, identity, and cultural affiliation as they age, in addition to having greater health and care demands. Thus, gaining insight into their experiences can help provide social and health services for older migrants. To the best of our knowledge, no study has investigated the experiences of cultural adaptation in older Turkish immigrants living in Norway. The focus was on better understanding the cultural adaptation of immigrants who came to Norway at a young age, from arrival until old age, and during the same period. In the present study, we aimed to explore the acculturation experiences of these long-term older Turkish immigrants living in Norway, how they interact and cooperate with members of Norwegian society and their experiences of life in society. The research question was: “How do long-term Turkish immigrants in Norway perceive their cultural adaptation from their arrival to old age?”

## Method

### Design

This study had a qualitative, explorative design and employed reflexive thematic analysis. It was prepared in line with the SRQR checklist (Standards for Reporting Qualitative Research) [37] (Supplementary File: SRQR Checklist). This study was conducted with Turkish immigrants of old age living in Norway. Data were collected using individual semi-structured face-to-face interviews between March and June 2023.

### Sample

The study sample consisted of 15 older Turkish immigrants aged 60 years and over. Participants were selected using the snowball sampling method [38]. Nevertheless, when we included participants, good variation was obtained in terms of sex and social background. The inclusion criteria were, in addition to being 60 years of age or older: being born in Türkiye, immigrating to Norway at a young age, and speaking Turkish or Norwegian.

The participants came from a small geographic area, specifically Norway, which includes the central city of Trondheim. The majority of participants continued to use Turkish culture and language since they lived in an area with a high concentration of Turkish immigrants. Norway, the destination of immigration in this study, provides high-quality social services to newly arrived immigrants.

Among the 15 older Turkish participants, 60% were women, 53.3% were between 60 and 64 years old, and no participant was older than 74 years. All participants were married and lived in Norway. Their period of residence in Norway was 30.5 ± 6.7 years.

### Data collection and instruments

Individual face-to-face semi-structured interviews were held in a quiet and comfortable environment in a place preferred by the participants, allowing them to express themselves freely. The duration of the interviews ranged from a minimum of 25 to a maximum of 60 min. A semi-structured approach provided flexibility for older migrants to articulate their unique experiences and perceptions without the constraints of structured methods or standard measurement tools used in quantitative research. In this study, an interview guide was developed by the authors in line with the purpose of the study, taking into account the questions in previous research literature [28, 39, 40] and focusing on the concepts of migration experience, adjustment difficulties, life in host country, social interaction, cultural perception, cultural identity (Supplementary Table: Interview Guide).

The interviews could be offered using Turkish or Norwegian language. All participants preferred using the Turkish language. The interviews were recorded. Field notes were taken from each participant after each interview. All interviews were transcribed verbatim by the second and third authors (BNT and SŞ) and checked for accuracy by the first author. No new codes were determined in the 15th meeting. The data obtained from the interviews were rich and sufficient to address the topic of interest [41].

### Data analysis

The data were analyzed using Braun and Clark’s reflexive inductive thematic analysis methodology [41]. According to Braun and Clark, a theme represents a repeated pattern of importance that indicates something essential within the data and is relevant to the research inquiry. It offers a foundational framework for comprehending participants’ experiences, interpretations, and realities [42]. This analytic approach consists of six steps: (i) becoming familiar with the data, (ii) creating codes, (iii) combining codes into themes, (iv) reviewing themes, (v) determining the importance of themes, and (vi) reporting findings. In the first step, data familiarization is initiated, followed by analysis. All audio recordings were transcribed and quality-assured (BNT/SŞ). The transcripts were then translated into Norwegian so that all authors could read and reread the transcripts to identify patterns of meaning, contradictions, and inconsistencies. Two researchers (AI/BNT) carefully read each transcript. In the second step, the first author regularly met the corresponding coauthors and systematically defined the meaning of the dataset. The data were reviewed sentence-by-sentence, and codes were generated. Codes that were named differently but had similar meanings were brought together, which simplified data organizing. In the third step, the codes and their meanings were grouped as concepts or constructs. The generated codes were combined into themes. In the fourth step, potential themes were reviewed again by two authors (AI/BNT) and continually transformed into patterns of meaning. They reviewed the codes and data to ensure that the themes were applied correctly. In the fifth step, the research team met face-to-face and online to discuss codes and themes and resolve disagreements. The themes were reviewed by all researchers, and their names were clarified. In the sixth step, the authors wrote the scientific study report. Illustrative quotations are provided to reinforce the analytical findings. Table 1 presents an example of the analytical process.

**Table 1:** Main themes, sub-themes and codes which were identified from interviews regarding adaptation to a new culture

Main theme	Sub-theme	Open codes
Encouraging Cultural Adaptation	• *Experiencing social equality*,* tolerance and economic prosperity*	Equal access to healthcare services Human rights and equality are respected High cultural level of society Sufficient income to meet needs
	• *Meeting supportive people and functioning welfare systems*	Language training as part of the start package Norwegians are helpful, tolerant, and compassionate Cultural sensitivity of healthcare professionals Access to social and health services in old age
	• *Learning and liking the culture and people*	Preference for Norwegian culture and lifestyle Participation in local holidays and celebrations Interest in local travel and leisure culture Embracing cultural differences
Restricting Cultural Adaptation	• *A continuing language barrier: struggling to communicate*	Difficulty expressing culture due to language Inability to explain symptoms to healthcare professionals Dependence on interpreters for communication Ongoing isolation linked to a lack of language skills
	• *Preserving* one’s *own culture and heritage*	Maintaining Turkish food, language, and traditions Living near or with Turkish relatives in the host country Limited interaction with Norwegians beyond greetings Maintaining traditional gender roles

The sociodemographic characteristics of the participants are presented in Table 2. This was not a linear process, and all authors contributed their cultural knowledge and backgrounds.

**Table 2:** The general information including the sociodemographic characteristics of participants

Participant No	Age	Sex	Education level	Marital status	Working status	How many years have you lived in Norway?	Family members with whom she/he lives	Norwegian speaking status	Can you read Norwegian?	How would you describe your Norwegian skills?
P1	70–74	Male	High school	Married	Retired	33 years	Spouse and children	Yes	Yes	Good
P2	65–69	Male	Primary school	Married	Retired	36 years	Spouse and child	Yes	Yes	Good
P3	65–69	Male	Secondary school	Married	Retired	36 years	Spouse	Yes	Yes	Excellent
P4	60–64	Male	Primary school	Married	Working	33 years	Spouse and child	Yes	Yes	Good
P5	65–69	Male	Secondary school	Married	Working	28 years	Children	Yes	Yes	Good
P6	60–64	Female	Primary school	Married	Not working and not retired	26 years	Spouse and children	Little	Yes	Good
P7	60–64	Female	Primary school	Married	Not working and not retired	24 years	Children	No	No	Very Poor
P8	70–74	Female	Primary school	Married	Retired	33 years	Spouse and children	No	No	Poor
P9	60–64	Female	Primary school	Married	Retired	31 years	Spouse and child	A little	A little	Middle
P10	65–69	Female	Primary school	Married	Retired	26 years	Spouse	Yes	Yes	Poor
P11	60–64	Female	Primary school	Married	Retired	29 years	Spouse	Yes	Yes	Middle
P12	70–74	Male	Primary school	Married	Working and retired	46 years	Spouse and child	Yes	Yes	Excellent
P13	60–64	Female	Primary school	Married	Working	17 years	Spouse and children	A little	Yes	Poor
P14	60–64	Female	Primary school	Married	Not working and not retired	26 years	Spouse and children	Yes	Yes	Good
P15	60–64	Female	Primary school	Married	Working	34 years	Spouse and child	Yes	Yes	Poor

### Preunderstanding

This qualitative analysis reflects the positionality, clinical and academic background, and cultural identity of the researcher. The first (AI) and the fourth researcher (SG), working in the field of public health nursing, conducted studies in the areas of older adults’ health, migration, and cultural care in Türkiye. The first researcher has 15 years of clinical and academic experience, and the fourth researcher has 40 years of clinical and academic experience. The second researcher (BNT) is a doctoral student in surgical nursing and the third researcher (SŞ) is a doctoral student in public health nursing. These researchers also have 9 and 6 years of both clinical and academic experience, respectively. The fifth researcher (ASH) works in public health and nursing and has published research on the health and quality of life of older people in Norway and immigrants. This researcher also has more than 40 years of clinical and academic experience and was born and lives in Norway. Except for the last author, all other researchers were born in Türkiye. All researchers have previously published qualitative research and have reflected on their positionality and previous experiences to take a more informed and comprehensive approach to data analysis.

### Ethics consideration

The need for ethical approval for this research project was waived by the Regional Committee for Medical and Health Research Ethics in Norway because it was not intended to generate new knowledge about health and diseases. This research project was registered and conducted by the protocol of the Norwegian Center for Research Data (ref. no. 170592). Written information in Turkish and Norwegian was given to potential participants. Additionally, the purpose of the study and its content were explained orally to the participants before providing written informed consent. Information about their right to not participate in the study and to leave the study at any time without giving any reason was given to all participants. The voice was recorded, but no other directly identifiable information was collected from the participants. Throughout the study, the researchers adhered to the principles of the Declaration of Helsinki.

## Results

This study generated two main themes and five subthemes regarding cultural adaptation to their host country: (a) “Encouraging cultural adaptation” including the subthemes: *experiencing social equality*,* tolerance*,* and economic prosperity; meeting supportive people and functioning welfare systems; learning and liking the culture and people*; (b) “Restricting cultural adaptation” including the subthemes: *a continuing language barrier: struggling to communicate; preserving* one’s *own culture and heritage* (Table 1).

### Main theme 1: Encouraging cultural adaptation

#### Experiencing social equality, tolerance, and economic prosperity

Almost all participants stated that Norway was excellent in terms of social equality, tolerance, and economic prosperity and encouraged their adaptation to the country they migrated to. It has been stated that there is equality in human rights in countries where migration occurs.Participants do not experience exclusion from Norwegian society. This makes it easier for people to fit in with the country and feel welcomed. Social equality in society contributes to adaptation; you were treated as fearful and equal to Norwegians by birth. One participant emphasized social equality by describing the specialist health care services where the economy and position of the person in need of services are irrelevant for treatment options or quality of care, “In Türkiye, to get good health care in hospitals, you have to go to private hospitals and money is needed for this, but in Norway, the president or anyone from the public receive equally good health care (at the same public hospital)” (P3, M, 36 years).

Another aspect contributing to adaptation in society was the high cultural level of the Norwegians. This was exemplified with expressions like people do not talk behind immigrants’ backs, do not belittle them, and respect the way participants dress (respect for veiled women). They stated that Norwegian managers/employees and local people were not excluded in any way in working life or society in Norway (P4, M, 33 years; P7, F, 24 years). Nevertheless participants usually did not socialize outside work or in leisure activities.

Furthermore, there is poverty in Türkiye, and that is a factor that makes life easier in the host country (P10, F, 26 years; P12, M, 46 years). One participant narrated the situation stating: “He said that he could easily buy the things he wanted with the salary he earned in Norway (P12, M, 46 years). Economic gains in the host country allowed them to adapt to a certain degree.

#### Meeting supportive people and functioning welfare systems

Support from the Norwegian authorities, local Norwegians, and healthcare professionals has been identified as a factor encouraging adaptation. Many participants stated that the Nation has public health and social services and financial support that covers all citizens. One participant stated that when he brought his wife and children to Norway, the National authorities and social workers provided them with accommodation and support in finding a job and buying a house (P1, M, 33 years). They were all given language training as a part of the start package in the new country. It was stated that the people make an effort to listen to them and that Norwegians are understanding, merciful, helpful, and tolerant. One participant explained that he was grateful to the Norwegian system and the Norwegian values and the help he got from people (P2, M, 36 years). Participants stated that healthcare professionals, in addition to providing medical treatment and care, are passionate, supportive, and want to adjust to patients and the next of their cultural values and traditions while hospitalized. One participant explained that the day her mother passed away, the nurses respected the culture of the patient’s relatives, were helpful and understanding to them and that she would never forget this moment (P9, F, 31 years). The wish to help people is not limited to healthcare professionals or professional helpers. Another participant stated that her neighbors helped her when she fell and that Norwegians were compassionate (P15, F, 34 years).

They came to the host country as economically migrant migrants, and the health and welfare system in old age contributed to making life in the host country acceptable and, to a certain degree, improving adaptation. It was stated that living conditions and retirement salaries in Norway are better in old age than in Türkiye. One participant stated that if he had been in Türkiye during his retirement period, his financial situation would have been lower, and his conditions were very different from those of his peers in Türkiye. The same participant stated that the Norwegian government provides financial and spiritual support (P1, M, 33 years). Some participants stated that the government’s social and health care support in old age is better than in Türkiye (P3, M, 36 years; P11, F, 29 years, P15, F, 34 years). A few participants also stated that health and social service support in nursing homes was good and that health and social experts cared for their children if they could not provide such care (P11, F, 29 years; P15, F, 34 years).

#### Learning and liking the culture and people

Some participants stated that they liked Norwegian culture more than Turkish culture, that they liked their lifestyle, and that their communication style was better than that of the Turks. Some Turkish immigrants stated that they easily adapted to Norwegian culture because they liked it. One participant explained that Norwegians have better conversational skills than Turks and are more polite (P5, M, 28 years). It has been stated that learning their language and learning about their special days/celebrations helps one get used to the culture and adapt (P2, M, 36 years). One participant stated that their lifestyle is very good, they love traveling, and grown-up children can easily spend time with their families inside and outside the house, go to restaurants or bars, and be treated equally with their parents (for example taking a glass of alcohol together), which would be rare in Turkish culture. In addition, this participant expressed that they celebrated Norwegian special days together (P4, M, 33 years). However, this participant was more of an exception to what most participants wanted.

### Main theme 2: Restricting cultural adaptation

#### A continuing language barrier: struggling to communicate

Almost all participants stated the greatest hindrance to cultural adaptation, especially shortly after migration. Some participants stated that in the beginning, they could not explain their cultural background and preferences to Norwegians because they did not know the Norwegian language. Moreover, they could not explain their symptoms and health problems to health personnel. The solution was to find someone who spoke a language (an interpreter) (P4, M, 33 years; P5, M, 28 years; P6, F, 26 years; P14, F, 26 years).

In the beginning, when men met others and socialized with others, it was hard. One participant shared how he could not explain himself and his beliefs to people: “I couldn’t explain to people why I didn’t eat pork or drink alcohol” (P4, M, 33 years). The possibility of traveling both locally and nationally could also be restricted, especially during the first migration period for all participants. They had problems using transportation because they did not know the language. One participant stated that he could not explain his route when he got on the bus. The same participant shared that in his home life, he did not have any problems (he spoke Turkish there), while finding work and working were problematic in the beginning because he had not learned the language. He also shared that knowing a certain level of English would have reduced initial language problems since most Norwegians, in addition to their language, speak English (P5, M, 28 years). One participant explained how she experiences language problems, “When a person does not know the language, the person looks deaf and dumb because they cannot answer the questions” (P14, F, 26 years). In the first period of migration, both genders experienced language difficulties. However, many female participants continue to experience language difficulties. They said they had housewife duties and did not have paid work outside the home at any time and thus were not exposed to the language much. Most of the male participants learned Norwegian to work outside the home before retirement.

#### Preserving one’s own culture and heritage

Many participants continued their own culture and did not integrate well with Norwegian society. Part of this may have been due to having relatives from Türkiye who had come with them or had already arrived; thus, they had Turkish society in their neighborhood. One participant stated that they continued to cook and eat Turkish food and maintained their culture and language as they had learned from childhood. The same participant stated that they maintained kinship relations within their family, which made it easier for them to practice their religious beliefs in Norway (P1, M, 33 years). Most participants lived in areas with other people of Turkish cultural heritage and common areas where family members lived. It was reported that it would be difficult to live in a host country in an area where there are no relatives. While more than half of the participants stated that their communication with Norwegians was no more than greetings, they also stated that they did not experience any problems being in Norwegian society because they had Turkish relatives in the area where they lived. They maintained their Turkish identity and culture by limiting their social activities with Norwegians.

Most of the participants explained that there were differences between Norwegian culture and Turkish culture, and they did not want to break away from their own culture. It was connected to value differences between the cultures, but also the lack of Islamic background. Many participants mentioned the Norwegian food culture including eating pork and also alcohol intake which is considered a sin in Turkish culture and Islam. One said explicitly that this is the reason they cannot adapt (P9, F, 31 years). Another participant stated that in Turkish culture, girls are not allowed to go out in the evening, but there are fewer such restrictions in Norway (P11, F, 29 years). One participant stated that she did not want to learn about Norwegian culture, that her clothing and lifestyle were different, and that she should continue in that way (P14, F, 26 years).

Preserving one’s own culture could also limit collaborations and friendships with migrants from other cultures that share Islamic beliefs. One participant explained herself like this: “Iranian, Iraqi, Syrian, they are all Muslims. Even though they are Muslims, I do not want my children to marry them. I want them to marry someone from Türkiye. I don’t like foreigners.” (P 15, F, 34 years).

Some of the participants point towards cultural differences in old age. They stated that Norwegians continue their working lives and live an active physical and social life in old age. They were looked upon as having a more dynamic life with less cultural restriction in how to live in old age. One participant said that immigrant Turks cannot keep up with this situation, and even that Turks live a life with death on the doorstep and have a stable sedentary life (P5, M, 28 years). It was found that most participants shaped their lives in line with their culture and raised their children in the same manner. Thus, when preserving their own culture, participants tried to teach Turkish culture and behavior to their children and grandchildren and expected them to be taken care of if necessary (P11, F, 29 years; P14, F, 26 years).

## Discussion

This study sought to investigate how older Turkish immigrants in Norway adapted to their new culture by examining how they saw the process from the time of their arrival till old age. Turkish immigrants who have been in Norway for a long time say that favorable aspects including economic growth, social equality, and tolerance promote cultural adjustment. However, communication problems and language impediments are the primary factors that make adaptation challenging. Due to their preference for maintaining their own culture, participants find it difficult to fully integrate into Norwegian society and integrate socially. When migrants get older, cultural differences become more noticeable, and they tend to adhere to their own cultural beliefs. The discussion section is presented according to the two main themes identified in the present study: (a) Encouraging cultural adaptation and (b) restricting cultural adaptation.

### Encouraging cultural adaptation

Factors that encourage the cultural adaptation of the participants include social equality, tolerance, and economic prosperity in the countries they immigrate to. In a study of Romani people who immigrated to European countries, the participants experienced they were appreciated and valued in the host country [8]. In a study conducted with Polish immigrants who immigrated to Norway, it was reported that immigrants had more time and money in the destination country, and the money they had was enough to buy many things [43], which is in line with the findings of the present study. Furthermore, our participants stated that they were treated equally with Norwegians and were not discriminated against due to nationality, age, and gender, which is also supported by the findings in the study about Polish immigrants to Norway [43]. However, studies in Austria and Finland report that immigrants are discriminated against and that stigma affects adaptation negatively [44, 45]. When people migrate with the wish for a better economic life, their adaptation to the destination country may become easier than when they immigrated for other reasons [46, 47]. In a study of immigrants from Nepal to Qatar, professional employment opportunities and higher earnings in the destination country provided hope for a better future [48]. Individuals in the present study migrated to Norway, where the economy and living conditions were better at the time of arrival. Economic and social welfare conditions in the host country may positively affect the economic and social adaptation of immigrants [47, 49], in fact, similar to the findings in the literature [46, 47], Turkish immigrants living in Norway, which has good economic and social welfare conditions, made similar statements.as the participants in the present study expressed.

Participants stated that they were grateful to social workers and the Norwegian government for providing social service support after immigration and that housing, language, work, and family reuniting encouraged adaptation and inclusion into the new society. The findings of the present study are supported by a study examining the experiences of Chinese immigrants to the UK, which reported that opportunities to find a home positively affected inclusion in society [50]. Receiving adequate and high-quality service from the government, voluntary or private institutions in the destination country positively affects their adaptation to the new country [51].

Getting accustomed to the culture one migrates to is encouraging adaptation. In the study examining the adaptation experiences of African immigrants (voluntarily or involuntarily) who lived in Australia long (up to 17 years), it was found they had good mental health and positive future expectations at the time of study participation [52]. Cultural integration results in positive mental health [53]. It has been stated that Chinese immigrants in South Korea adapt to the cultural values of the destination country, and define themselves as South Koreans, and that adaptation also includes psychological and social changes [54]. The literature [52–54] and study findings are similar, suggesting that acculturation is easier when immigrants appreciate, embrace, and feel a connection to the culture of their host country. However, only a small number of current sales indicate that they desire to adapt to or become part of the host country’s lifestyle.

In a systematic review examining the experiences resulting from the health care of individuals with immigrant backgrounds, it was found that healthcare providers viewed those with immigrant backgrounds as “the other” and did not feel confident or equipped to treat these “others” [55]. Contrary to this review, all participants in the present study reported that the Norwegians and their government are good in terms of human rights and equality, and they had never experienced any discrimination. Instead, they stated that Norwegian healthcare professionals (doctors, nurses) provide high-quality treatment and care, including respect for the cultural values of immigrants. Furthermore, although the quality of health services in old age was reported to be high, most participants did not wish to receive care support from outside the family if they have functional disabilities and care needs in the future. Therefore, understanding the cultural adaptation processes of older immigrants who may eventually experience cognitive and functional impairment is crucial for nurses in order to create care plans that are supportive, considerate of the patient’s cultural values, and cognizant of their cognitive needs [56, 57].

### Restricting cultural adaptation

The most important factor that makes cultural adaptation difficult is the language barrier. All participants emphasized that not knowing their language was the biggest problem they encountered. It has been stated that not knowing the language causes problems in accessing health services, using public transportation, and preventing communication with Norwegians. It was reported that the language problem was greater when they first arrived; however, the language problem persists due to the less participation of immigrant women in Norwegian society. In addition, all the female participants were at the primary school level, but the education level of the male immigrants was not very high. Language training is offered by the Norwegian government to all, but several do not attend [58]. In a study conducted with immigrants in Brazil and France, psychological distress was experienced after migration because of the new language and cultural environment [59]. In a study examining the experiences of those who went to England and Norway, some stated that they had problems communicating with people and finding friends because they did not know the language well, that misunderstanding what was said led to mistakes, and that not knowing the language caused them to feel like they were disabled in a way [60]. Studies conducted with Turkish migrants in Germany and Belgium also report that migrants face language barriers [61–63]. This can make it difficult for them to integrate into the host society and access health and social services [64]. This experience agrees with the experiences that all the participants had when they came from Türkiye to Norway. However, after many years (mean 34 years) in Norway, female immigrants especially speak and understand Norwegian poorly, which limits their adaptation. Language has been a problem mentioned in most studies on immigrants [65–67]. Nurses can help migrants overcome language barriers and provide care that supports social integration. Furthermore, cultural awareness and sensitivity enable nurses to more effectively support migrants’ cultural adaptation. Moreover, providing free language education to immigrants to Norway is an excellent opportunity for them. All recipient countries need to implement such initiatives to address language problems.

Protecting immigrants’ culture is important for their well-being [68], but as we find in the present study, it may prevent cultural adaptation. Immigrants who came to Norway for economic reasons years and years ago still want to feel Turkish in the Norwegian surroundings and preserve their values, culture, and language, as well as old age. This is possible and convenient because they live in areas with Turks and relatives. According to the participants, there are large cultural differences between the Norwegian and Turkish people, not only in language but also because they experience people from countries with Islamic beliefs to differ considerably in values and see them as foreigners. In line with our findings, a recent study on labor migration found that individuals maintained their cultural identity and continued to use their language to do so [69]. An Austrian study involving young Turkish immigrants sought to both maintain their childhood culture and adapt to Austrian society [70]. Immigrants protect and maintain their culture to create a safe environment [71]. This research indicates that Turkish migrants uphold a strong connection to their cultural identity and community, which can enhance psychological well-being while hindering broader social integration. These results align with those documented in existing literature. Additionally, this research reveals that as individuals age, cultural dissonance tends to increase, leading them to gravitate more towards familiar cultural norms and practices, consistent with studies involving older migrants from other countries. Therefore, this research reflects broader transnational adaptation patterns that have been observed, particularly in older migrant populations who have lived abroad for significant periods. It is crucial for nurses to comprehend these cultural attachments and offer considerate, encouraging care since migrants’ attempts to maintain their culture enhance their psychosocial security and sense of belonging. As a result, it is advised that nurses improve their intercultural communication abilities and create customized treatment plans that consider immigrants’ cultural values [72].

### Strength and limitations

One strength that should be mentioned is that the participants could be interviewed using their native language to express their experiences, but if some wanted to be interviewed in Norwegian, it was an option. The presence of researchers with both elaborative knowledge of Norwegian and Turkish society and culture contributed to a balanced and many-faceted understanding of the interviews and enriched the analytic process. The second and third researchers who collected data had previously conducted qualitative research and both lived in Norway for 6 months as part of a postgraduate study mobility. Thus, they had the chance to get to know both Turkish immigrants and Norwegians in Norway. The last researcher supervised these two researchers during the data collection process. The participants came from a limited area, namely Norway, a major city in the middle of the country. We cannot guarantee that Turkish immigrants in this region will not differ in their experiences regarding cultural adaptation from those of Turkish immigrants in other parts of Norway or whether we have included a sufficiently diverse group of participants. One limitation is that none of the oldest Turkish immigrants volunteered for the study. However, 15 participants of both genders and social backgrounds were included, and in the final interview, no new codes emerged.

## Conclusion

Turkish immigrants indicated that social equality, tolerance, and economic prosperity in Norway had a positive impact on their adaptation process. The effective systems for social assistance in Norway, the support from the local community, and the attentiveness of healthcare professionals were also highlighted as significant factors that aided in adaptation. Some individuals mentioned that their integration was smoother because they appreciated and embraced the local Norwegian culture. However, the language barrier presented a major challenge for communication and accessing healthcare services, particularly in the initial stages of migration, and this issue continued for an extended period, especially among female participants. Most respondents expressed a preference for maintaining their own cultural identity due to the differences in religious and cultural values, which complicated their full integration into Norwegian society. Healthcare professionals ought to cultivate communication skills that are culturally attuned to assist Turkish immigrants residing in Norway in navigating language obstacles and offer interpreter assistance when necessary. Additionally, they should design personalized care plans that honor the cultural values of patients and implement methods that ease the integration process while encouraging immigrants to preserve their cultural identity. To tackle the difficulties encountered by Turkish migrants in Norway, public institutions and local authorities should provide free and easily accessible language training programs while also developing social inclusion initiatives that enhance language learning opportunities, particularly for women. It is essential that cultural competency training is made compulsory for health and social service professionals, alongside the need for ongoing support for those working in this sector. Furthermore, cultural counselling services should be established, and programs aimed at social integration and psychosocial support for migrants should be planned and executed in collaboration with local governments, healthcare organizations, civil society groups, and representatives from migrant communities.

## Supplementary Information

Below is the link to the electronic supplementary material.

Supplementary Material 1

Supplementary Material 2

## Data Availability

The data that support the findings of this study are available from the corresponding author upon reasonable request.

## Declarations

### Author information

This qualitative analysis reflects the positionality of the researchers. The first (AI) and fourth researchers (SG) were experienced scholars with a PhD in public health nursing, having conducted studies in Türkiye on older adults’ health, migration, and cultural care. The second and third researchers (BNT and SŞ) were doctoral students in the Department of Public Health and Nursing at the Norwegian University of Science and Technology University during the study. The fifth researcher (ASH) was a nursing professor in Norway with published research on older adults’ health, quality of life, and migrants. ASH also supervised the two doctoral students. All researchers had prior experience in conducting and publishing qualitative research and reflected on their positionality and previous experiences to adopt a more informed and comprehensive approach to data analysis.
