# Supplementary material for: Cultural adaptation experiences of long-term older Turkish immigrants: a qualitative study
Source: BMC Nurs. 2025 Sep 26;24:1197. doi: 10.1186/s12912-025-03848-6 (PMC12465484; doi:10.1186/s12912-025-03848-6)
Supplement: Supplementary file 1 — Supplementary Material 1 [file 12912_2025_3848_MOESM1_ESM.docx]

**Supplementary Table:** **Interview Guide**

| *The interview guide and its four main questions.*   1. Can you share your experiences of being an immigrant in Norway, from your arrival until old age? 2. As an immigrant living in Norway, how would you describe your participation in society, collaboration, and communication with individuals in Norwegian society? 3. What are your thoughts about the culture of individuals in Norway compared to your own culture? 4. Can you share your experiences of cultural adaptation during the migration period and in your old age? |
| --- |
